# Leveraging brief annual pauses in implementation: Using a rapid qualitative approach to inform iterative planning and adaptation of a school-based asthma program

**DOI:** 10.1017/cts.2026.10730

**Published:** 2026-03-25

**Authors:** Julia Reedy, Nicole M. Wagner, Amy G. Huebschmann, Avery Schaefer, Anowara Begum, Rachel Armstrong, Michaela Brtnikova, Melanie Gleason, Stanley Szefler, Lisa Cicutto, Sarah E. Brewer

**Affiliations:** 1 Adult and Child Center for Health Outcomes Research and Delivery Sciences (ACCORDS), University of Colorado Anschutz Medical Campus School of Medicine, USA; 2 Internal Medicine, UCHealth, USA; 3 Division of General Internal Medicine, University of Colorado Anschutz Medical Campus School of Medicine, USA; 4 Ludeman Family Center for Women’s Health Research, CU Anschutz Medical Campus, USA; 5 Department of Pediatrics, University of Colorado Anschutz Medical Campus School of Medicine, USA; 6 Breathing Institute, Children’s Hospital Colorado, USA; 7 National Jewish Health, USA; 8 College of Nursing, University of Colorado Anschutz Medical Campus, USA; 9 Clinical Science Program, University of Colorado Anschutz Medical Campus, USA; 10 Department of Family Medicine, University of Colorado Anschutz Medical Campus School of Medicine, USA

**Keywords:** Rapid qualitative analysis, practical robust, implementation, and sustainability model (PRISM), asthma, pediatrics, school-based intervention

## Abstract

**Background:**

Asthma is a prevalent chronic pediatric condition associated with significant health disparities. The Better Asthma Control for Kids (BACK) program aims to reduce asthma disparities and improve asthma control for children in high-need schools in four regions of Colorado.

**Methods:**

We conducted in-depth, semi-structured interviews (Dec 2023–June 2024) with key roles involved in BACK including school nurses, asthma navigators, and caregivers of participating students with asthma. Interviews and rapid qualitative analysis were informed by the Practical Robust, Implementation, and Sustainability Model (PRISM). We gathered perspectives, feedback, and recommendations about BACK to inform adaptation of the intervention and implementation strategy packages.

**Results:**

Participants (*n* = 39) included 6 asthma navigators, 17 school nurses, and 16 caregivers. Four overarching themes emerged: 1) perceived benefits of the BACK program, 2) challenges with school nurse engagement, communication, and perceptions of BACK, 3) difficulty with identification, documentation, and enrollment of students with asthma at the beginning of the school year, and 4) mismatches in program scope and alignment with school and family contexts.

**Conclusion:**

Identifying key challenges and participant recommendations supported the research team’s “adapt and tailor to context” strategy in annual rapid evaluation and planning cycles. Obtaining feedback from program adopters, implementers, and recipients led to complementary recommendations to improve program delivery and user experiences. This approach may be transferable to other implementation-effectiveness trials, particularly those in schools or with other natural pauses that facilitate annual iterations in program delivery.

**Trial registration:**

Clinicaltrials.gov identifier NCT06003569, registered on August 22, 2023, https://clinicaltrials.gov/study/NCT06003569

## Introduction

Asthma is a prevalent chronic condition in children, disproportionately impacting those living in communities with limited resources and access to healthcare [1]. Asthma disparities can, in part, be explained by differences in social determinants of health (SDOH) including access to healthcare, transportation, and insurance [2–8]. For the last two decades, our school-based asthma programs in the Denver metropolitan area have been effective at reducing asthma exacerbations, school absenteeism, asthma-related hospitalizations and emergency department visits, and steroid use [9–12]. These programs utilize community asthma navigators to facilitate care coordination and case management for students with uncontrolled asthma and their families, including addressing unmet SDOH needs. Education provided by asthma navigators has improved asthma knowledge and control, inhaler technique, and self-management skills leading to improved quality of life for students and families [9–12]. Building off the success of our Denver-based programs, we connected with school and public health leaders across Colorado to identify other school districts interested in adapting this program’s core components to their local context.

We utilized community input to a) adapt core program functions to rural and semi-urban communities across Colorado with larger minority and low socioeconomic populations and b) design implementation strategies to tailor methods of delivering this program to the contextual priorities, resources, and needs of these settings [13]. This adapted program, Better Asthma Control for Kids (BACK), centers around six core functions: 1) Identify children with asthma; 2) Assess asthma control to confirm poorly controlled asthma (eligibility for BACK); 3) Ensure completed asthma care plan and rescue inhaler are at school; 4) Provide tailored instruction to develop student’s and caregiver’s asthma knowledge and self-management skills; 5) Provide asthma case management and care coordination across family, school, and health care providers; 6) Conduct a standardized SDOH assessment and connect families to resources to support their SDOH needs [13].

As the implementation science field continues to evolve to incorporate adaptive implementation strategies and program designs supporting a more rapid pace of implementation [14], specific case studies of ways to use rapid evaluations of context to support adaptation are needed. This type 2 hybrid implementation-effectiveness trial of the BACK intervention [13] acts as a case example, utilizing an “adapt and tailor to context” implementation strategy [15]. This “adapt and tailor to context” strategy is applicable across both the standard implementation strategy package (BACK-S) and the enhanced strategy package (BACK-E) which adds network weaving between BACK implementers, adopters, and recipients. Adaptations of the current intervention and implementation strategies must meet context-appropriate needs while maintaining fidelity to core program functions. In the present manuscript, we describe how we operationalized rapid qualitative evaluations from multiple program partner and participant perspectives within a limited 2-to-3-month time frame to support our “adapt and tailor to context” implementation strategy [15]. This approach may be transferable to other implementation-effectiveness trials, particularly those in schools or with other natural 2–3 month pauses that facilitate annual iterations in program delivery.

## Materials and methods

### Study design

The BACK hybrid implementation effectiveness trial launched in August 2023. In brief, nurses from eligible schools who agree to adopt BACK were either randomized to a 1-year wait list control, a 2-year standard implementation strategy package (BACK-S), or a 2-year enhanced implementation strategy package (BACK-E). Implementers are asthma navigators who work with nurses in their study arm to deliver BACK to student and caregiver recipients (see [13] for further details).

The “adapt and tailor to context” strategy required an annual rapid evaluation and planning cycle whereby the study team reviewed preliminary qualitative data and process measure findings to guide priority recommendations for modification in the subsequent school year. This rapid qualitative research was designed to identify opportunities to refine the implementation strategies being tested within the trial. While this paper focuses on the adapt and tailor implementation strategy, a brief overview of all strategies is included in Table 1.

**Table 1.** Overview of implementation strategy packages and key roles

Participant group	BACK program implementation role	BACK-S implementation strategy package^a^	BACK-E implementation strategy package^a^
School nurse	Adopter	*Adapt and tailor to context^c^* Training Facilitation Engage Families Develop educational materials Develop partner interrelationships Support clinicians (school nurses and health care providers)	All BACK-S strategies Network weaving between adopters, implementers, and recipients
Asthma navigator	Implementer		
Caregiver	Recipient		
Child with uncontrolled asthma^b^	Recipient		

a *Note:* BACK-S and BACK-E implementation strategies were delivered to each school nurse and offered to eligible students in their school during the Implementation Phase of the trial from 2023–2026. See protocol manuscript for further details [13].

b Child data not included in this manuscript.

c Adapt and tailor to context implementation strategy is primary focus of present manuscript.

For this work, we used the integrated Practical, Robust Implementation and Sustainability Model (PRISM) that includes the RE-AIM (Reach, Effectiveness, Adoption, Implementation, and Maintenance) outcomes and contextual determinant domains that influence RE-AIM outcomes [16]. PRISM was used to guide planning, implementation, and sustainment across the research life cycle of this trial through consideration of multiple partner and participant perspectives: program implementers (i.e., asthma navigators), program adopters (i.e., school nurses), and program recipients (i.e., families of children enrolled in the BACK program) [16]. According to PRISM, the impact or value of the program is defined through evaluation of RE-AIM outcomes [17–19]. This manuscript focuses on the annual data collection and analysis that precedes the rapid evaluation and planning cycle. PRISM guided this annual data collection and analysis process by prompting consideration of contextual determinants that influence successful implementation: recipient/deliverer perspectives and characteristics, implementation and sustainability infrastructure, and external environment [16,18,20].

We chose to a conduct a rapid qualitative analysis [21–23] using a matrix approach due to the need to collect, analyze, interpret, and apply the results between participating schools’ academic years in a limited 2-to-3-month time frame. Rapid analysis allows for a rigorous and pragmatic evaluation of the BACK program on a short timeline to inform the subsequent iteration of the program for the next school year. While traditional qualitative approaches often prioritize a theoretically rich and broad exploration of data, rapid analysis offers a rigorous approach to in-depth understanding of specific concepts when the scope of data is narrow and data collection and analysis are highly structured [24,25]. Our study is well-positioned for a rapid analytic approach due to the specificity of our research question, structured data collection and analysis based on PRISM constructs, and the need for a rapid turnaround to support iterative intervention and implementation planning, recruitment, and partner retention for the upcoming school year. This research was approved by the Colorado Multiple Institutional Review Board and follows the consolidated criteria for reporting qualitative research (COREQ) [26].

### Setting and sample

While this study is being conducted in four regions across Colorado, this manuscript presents findings from the three regions receiving BACK in the first year of the trial (2023–2024 school year): Pikes Peak area, Lower Arkansas Valley, and Morgan/Weld. These regions were selected for study inclusion based on factors contributing to asthma disparity such as rurality, rates of free-reduced lunch (>30%), and high proportions of non-white students.

Study participants for annual program evaluation and planning included three key groups of individuals involved with program delivery or participation: school nurses, asthma navigators, and caregivers of students with asthma. All school nurses supporting at least one school receiving the BACK program (*n* = 31) were eligible for inclusion and contacted about interview participation. Asthma navigators responsible for program delivery (*n* = 6) were invited to participate in an interview. Caregivers were eligible for interview if 1) they completed their participation in the BACK program and 2) either did not complete or indicated they were open to interview participation on their end-of-year survey (*n* = 39). All school nurses, asthma navigators, and English-speaking caregivers were recruited in English; Spanish-speaking caregivers were recruited in Spanish with support from Spanish-speaking asthma navigators; however, none completed interview participation. Participants were purposively sampled by region for each role. Participant recruitment continued until all eligible participants had received our maximum number of outreach attempts or until our target sample for a given region (*n* = 8) was reached. All participants consented to study participation before or at the time of their interview.

### Data collection

Our rapid qualitative approach was guided by PRISM and sought to elicit novel data on the contextual evolution and mismatches in the fit of BACK to help inform appropriate adaptation. Our interview guides were developed by qualitative and dissemination and implementation experts (SEB, JR, AGH, NMW) and explored PRISM framework domains from each partner’s perspective. Operationalized for this project, these domains and constructs include primary PRISM RE-AIM outcome metrics of Reach (and the related outcome of retention) and Implementation (fidelity and implementation quality), the PRISM determinants of multi-level characteristics and perspectives on the intervention (including feasibility, acceptability, appropriateness, and perceived effectiveness), implementation and sustainability infrastructure, and internal and external environment. For example, to solicit information about school nurse perspectives of the intervention and its perceived effectiveness, we asked “What impact do you think the BACK program has had on kids with uncontrolled asthma?” Full interview guides are provided in Supplements 1–3. A different interview guide was developed for each group of participants (i.e., BACK adopters (school nurse), implementers (asthma navigators) or recipients (caregiver)), exploring key domains specific to the components of the intervention or its delivery. In-depth semi-structured individual interviews were conducted either virtually or by phone and were audio recorded. School nurse and caregiver interviews lasted approximately 30 minutes; asthma navigator interviews lasted approximately 60 minutes. One trained qualitative researcher (JR), without any prior relationship to study participants, was responsible for conducting all interviews. Interviewees were compensated for their participation. Following each interview, a detailed interview summary was completed, mapping participant responses across framework domains and documenting a reflection on the interview including potential interviewer biases or assumptions.

### Data analysis

Interview recordings were professionally transcribed and de-identified. Two researchers (JR, AS) reviewed each interview summary, cross-checking against the audio recording or transcript to ensure accuracy. Our rapid approach utilized a matrix analysis to condense findings with a separate matrix for each participant group. The columns of each matrix contained priority PRISM framework constructs specific to that participant population. Across the rows of each matrix, data from interview summaries were synthesized for each priority construct and across participants within each group. Three researchers (JR, AS, SEB) then met to discuss summaries and draw connections across participant groups. Illustrative quotations were pulled from transcripts and included in a presentation of findings to our Community Advisory Board Members (which include nurse and caregiver representatives) as a form of member-checking prior to sharing back with the larger research team.

The rapid qualitative analysis following year 1 of study implementation (2023–2024 school year) focused on the contextual factors predicting our study’s primary implementation outcome of equitable student reach and retention, as well as perceptions of how the BACK program impacts student’s asthma control (primary health effectiveness outcome), program feasibility, acceptability and appropriateness (PRISM multi-level perspectives on the intervention that are key influences of adoption, implementation, and maintenance/sustainment), and PRISM multi-level perspectives on the quality of the intervention (implementation fidelity). Using PRISM as a structure to guide and narrow the scope of our qualitative analysis allows for the identification of key contextual strengths/facilitators and ongoing barriers/challenges to BACK that warrant adaptation to promote successful program implementation.

### Planned use of qualitative findings to inform the annual rapid evaluation and planning cycle

To act rapidly on qualitative findings, our study team used the following process to evaluate and plan the subsequent year’s implementation: 1) Our research team, comprising pulmonary providers, asthma content and school-based intervention experts, qualitative and quantitative methodologists, and dissemination and implementation scientists convened at an annual retreat; 2) qualitative findings were reviewed and discussed, in conjunction with other data sources (e.g., process measures of representativeness, periodic reflections with asthma navigators, school nurse learning communities) and considered in light of major observations by the study’s working groups, 3) the team used nominal group technique to brainstorm and prioritize the top 2–3 options to adapt the current study activities to address each contextual challenge. Following the study retreat, research working group teams (e.g., school planning, implementation, data analysis, study leadership) reviewed the priority recommendations to identify which adaptations were feasible based on the following criteria: a) fit with the core function of the existing intervention and implementation strategies [13], b) no contamination of the enhanced implementation strategy package (BACK-E) as compared to the standard implementation strategy package (BACK-S), c) financially viable given current research budget and feasible to sustain following completion of the research study, and d) deemed appropriate by state and regional study Community Advisory Boards.

## Results

We conducted a total of 39 interviews throughout the first year of the BACK program including 17 school nurses (RN) between December 2023 and February 2024, 6 asthma navigators (AN) in May–June 2024, and 16 caregivers (CG) in June 2024. Across all participant roles, we engaged 19 participants from the Pikes Peak area (PP), 12 from Morgan/Weld (GWM), and 9 from the Lower Arkansas Valley (LAV); findings did not differ substantially by region. One asthma navigator supported both the PP and LAV regions. All participants had a language preference of English and self-identified as female. Interview participant demographics are described in Table 2.

**Table 2.** Participant demographics

Variable	Pikes peak area	Morgan and weld counties	Lower arkansas valley	Total
**School nurses**	**8**	**7**	**2**	**17**
**Sex**				
Female	8	7	2	17 (100%)
**Asthma navigators**	**3**	**2**	**2**	**6^a^**
**Sex**				
Female	3	2	2	6 (100%)
**# Enrolled students**	46	29	11	86
**Caregivers**	**8**	**3**	**5**	**16**
**Caregiver sex**				
Female	8	3	5	16 (100%)
Caregiver race*				
Asian	0	0	1	1 (6%)
Black/African American	2	0	1	3 (19%)
Hispanic/Latino	5	1	2	8 (50%)
White	2	2	3	7 (43%)
**Caregiver age**				Median: 32 Mean: 33.4
**Student sex**				
Female	3	2	3	8 (50%)
Male	5	1	2	8 (50%)
Student race*				
Asian	0	0	1	1 (6%)
Black/African American	4	0	1	5 (31%)
Hispanic/Latino	4	1	2	7 (43%)
White	2	2	4	8 (50%)
**Student age**				Median: 8 Mean: 7.43
Insurance*				
Medicaid	5	1	3	9 (56%)
Child Health Plan Plus	1	2	2	5 (31%)
Private	4	0	1	5 (31%)
Uninsured	0	1	0	1 (6%)

*Note*: *Participants able to select all that apply.

a One navigator supported both the Lower Arkansas Valley and Pikes Peak regions.

Our rapid qualitative analysis led to the identification and development of four key themes. Participants described 1) perceived benefits of the BACK program 2) challenges with school nurse engagement, communication and perceptions of BACK, 3) difficulty with identification, documentation, and enrollment of students with asthma at the beginning of the school year, and 4) mismatches in program scope and alignment with school and family contexts. Illustrative quotations for each theme are included in Table 3.

**Table 3.** Quotation table

Theme	Supporting quotations
*Theme 1: perceived benefits of the BACK program*	*“I felt more comfortable letting my daughter go other places without me being around because she was confident and has the skills to be able to do her inhaler without me being there. She knew when to speak up and say I need my inhaler before things got too bad. And then she was also able to give it to herself and not have to rely on me to do it if I’m not around.” CG_PP5* *“I think it helps a lot […] there were lots of oohs and aahs teaching both students and the families. A lot of thankfulness from them, like, “Oh I didn’t know that. Thank you so much. It’s helping.” AN1* *“[BACK] has definitely helped with time management and havin’ someone else who will come and do the education piece with kids” RN_LAV1*
*Theme 2: challenges with school nurse engagement, communication, and perceptions of BACK*	*“When we first heard about [BACK] […] it was kind of irritating because it was implying that we weren’t doing enough on our own.” RN_GWM4* “*The most difficult part definitely was communication with the nurse, and some of ‘em were really responsive. Some of ‘em were not […] there was a lack of continuity in the communication between them and myself” AN6*
*Theme 3: difficulty with identification, documentation, and enrollment of students with asthma at the beginning of the school year*	*“One of our directors said, it’s like drinking out of a fire hose and I’m like, “That is exactly, exactly how the beginning of the school year feels” RN_GWM6* *“As far as communication is, just parents not getting back. I don’t blame them. I’m calling on my cell phone. I’m a person that they have not heard of before talking about their child and the school that their child goes to.” AN4* *“When I first heard about [BACK], it was briefly, so I wasn’t sure […]I tried googling about it before we got contacted, and I would just put the information being pushed out. There was nothing that came up.” CG_PP8*
*Theme 4: mismatches in program scope and alignment with school and family contexts*	*“I figured it’d be a bigger thing with the asthma navigator, the families and myself […] a grander scale of kiddos with the education and whatnot.” RN_GWM1* *“Everyone had different pieces of information like health—the nurses had […] the most information and working with health techs who are just like, ‘Who are you? Why are you here? What is my role with you?’ Front desk, principals, everyone was so—had just a different view of the program.” AN3*

### Theme 1: perceived benefits of the BACK program

Interview participants across roles described myriad benefits of the BACK program. This includes benefits for students, caregivers, and school nurses, each playing a key role in comprehensive and effective asthma management. Participants saw improvements in student self-advocacy, asthma self-management, improved student inhaler technique, and overall improved asthma knowledge. Instead of primarily relying on others, students were better able to recognize early asthma symptoms and communicate their needs which in turn makes caregivers more comfortable and confident in their child’s health and safety.

At baseline, prior to BACK participation, students had significant variability in their asthma; however, for many, improved self-management, inhaler technique, and asthma knowledge has led to overall improved asthma control. For one parent whose child had “very uncontrolled” asthma, they were “controlled enough that [my child] understands what is happening […] and what he can do to help prevent the attacks.” (CG_PP6). These perceived benefits are indicators of program effectiveness, a primary RE-AIM outcome. Benefits for students extended beyond the physical management of asthma and include multiple social benefits. Students were more able to participate in activities from which they had previously been restricted. Additionally, the BACK program made students feel special “in a positive way” (CG_PP5) because of their asthma; “it was nice to see happiness from her, especially because she feels so left out with having asthma.” (CG_GWM1).

A large component of the BACK program is engaging caregivers to improve their child’s asthma self-management – this relates to the overarching BACK core functions of asthma case management, care coordination and education [13]. Participants described benefits for caregivers including improved knowledge of and confidence in managing their child’s asthma, and improved awareness of steps to take during asthma exacerbations. While some caregivers felt knowledgeable and comfortable from the outset, others were “new to asthma” (CG_GWM3) and benefitted from education about “the correct times to be taking [medication]” (RN_GWM3) and “what steps [caregivers] needed to follow to prevent [an asthma exacerbation] from getting worse.” (CG_PP1). School nurses also felt that the BACK program benefited them by supporting their needs for effectively caring for students with asthma while at school. Many school nurses described feeling “spread thin”(RN_LAV2) and having very limited time to provide in-depth asthma education to students. Nurses appreciated that the BACK program offered student education, while also providing additional resources that can be leveraged to further support asthma management. These positive perceptions of BACK from both caregiver and school nurse perspectives may act as drivers for broader program adoption and reach in future years, another key implementation outcome.

### Theme 2: challenges with school nurse engagement, communication, and perceptions of BACK

At the time of their interviews, many school nurses had very limited experience with the BACK program and described common misunderstandings about their role in BACK. Lack of program familiarity from school nurses was largely due to challenges with initial program rollout, delays in starting the program, limited student enrollment, communication struggles internal and external to schools, and school district organization. In districts where school nurses support and split their time between multiple schools, they “don’t really have a lot of firsthand knowledge of [the BACK program]” (RN_PP1). Additionally, for some districts “getting all the approvals needed [caused] a big delay with [program startup] which set the tone for the rest of the year, it was a domino effect.” (AN4). Some school nurses felt their limited engagement to be a result of small enrollment numbers with student eligibility limited to those with uncontrolled asthma. For some schools, engagement occurred at the district level, with the BACK program “presented to the district” followed by a “top-down” (RN_PP4) approach to inform school nurses.

School nurses reported feeling there was limited transparency at the outset from the districts which impacted their level of involvement early in the program. This generated some misunderstandings about the role of school nurses in the BACK program. These misunderstandings contributed to communication challenges between school nurses and asthma navigators who together form the core of the program. At first, some nurses were frustrated with the program, feeling the BACK program would add to their already burdensome workload. Another nurse initially felt the BACK program was “irritating because it was implying that [school nurses] weren’t doing enough on [their] own.” (RN_GWM4). Other nurses were more indifferent to the program due to a lack of “understanding totally what [the school nurse] role is and the district’s role versus the asthma navigator’s” (RN_LAV1). Limited knowledge of the program and role misunderstandings contributed to communication difficulties between asthma navigators and school nurses. Some school nurses “didn’t answer [asthma navigator] emails at all. They were just ghosts” (AN1), essentially blocking student enrollment in the BACK program at those schools.

### Theme 3: difficulty with identification, documentation, and enrollment of students with asthma at the beginning of the school year

Several challenges with recruitment processes, communication, and reach at the beginning of the school year impacted student enrollment. School nurses described the start of the school year as hectic, overwhelming, and chaotic as they obtained all the proper medical and medication documentation from families to effectively care for students with chronic illnesses. According to one nurse, “The beginning of the school year feels […] like drinking out of a fire hose” (RN_GWM6); therefore, adding screenings for asthma, and coordination with the asthma navigator was difficult. The BACK program forced school nurses to prioritize care plans for students with asthma curtailing attention they could give to other high-priority chronic conditions, such as epilepsy, allergies, and diabetes. Multiple competing priorities at the start of the school year limited school nurse ability to outreach to families of students with asthma and contributed to some delays in enrollment.

Family communication with asthma navigators and lack of knowledge of the BACK program also posed challenges with recruitment and enrollment. The unclear role of the asthma navigator and their ambiguous relationship with the schools made some families wary and others choose not to respond to outreach attempts. Communication and subsequent enrollment were further inhibited as families had difficulty finding information about the BACK program and its legitimacy online. Lack of knowledge, uncertainty, and wariness of the program and outreach from an unknown navigator likely contributed to reduced enrollment constricting the potential reach of the BACK program. While the BACK program successfully enrolled some Spanish-speaking families, enrollment of participants with a language preference other than English was more limited.

### Theme 4: mismatches in program scope and alignment with school and family contexts

While school nurses, asthma navigators, and caregivers all viewed the BACK program positively, they identified areas that could be adapted to improve program fit and acceptability. Asthma navigators described feeling as though there was too much repetition between the three visits with caregivers and felt “asking different questions would be better for the visit” (AN2). School nurses were surprised by the scope of the study which limited student participation in the BACK program to those evaluated as having uncontrolled asthma. With some nurses having only a few students enrolled, some thought it was “gonna be a grander scale of kiddos” (RN_GWM1). One nurse said, “There were a couple of kiddos I was a little bit surprised that did not qualify for the BACK program” (RN_GWM2), others echoed this saying some of these students who come most frequently to the school health office were not eligible. Lastly, school nurses and asthma navigators reported that other school staff, including unlicensed assistive personnel (UAPs), teachers, front desk staff, and coaches, had minimal engagement and limited knowledge of the BACK program. With school personnel having differing levels of awareness and understanding of the program, navigators were questioned about their role within the school and if there was leadership approval, the purpose of their presence in schools, and how they interact with students. This impacted perceptions of the program from broader school staff and created difficulties for asthma navigators working to integrate with school systems and deliver the BACK program in the school setting.

### Adaptations addressing qualitative findings

Primary challenges described in our themes have influenced several adaptations for Year 2 of this hybrid-effectiveness trial. Adaptation examples, anchored by ERIC implementation strategies [27], a summary of primary challenges, and key findings are outlined in Table 4.

**Table 4.** Thematic drivers of BACK adaptations

Theme	Key finding prompting the need for adaptation	PRISM domain	Example Adaptation (ERIC implementation strategy*)
*Theme 1: perceived benefits of the BACK program*	N/A – these were positive benefits that did not require adaptations	Organizational perspective and participant perspective	N/A
*Theme 2: challenges with school nurse engagement, communication, and perceptions of BACK*	Program misperceptions among nurses and unclear expectations for nurse role	Organizational perspective- relationship and communication with adopters	Clearly defined the role, expectations, and BACK program purpose in introduction to school nurses (**Support clinicians (nurses)** and **Develop stakeholder interrelationships** in the implementation effort*)*
	Limited knowledge of the BACK program and its potential impact on students	Organizational perspective- ability to observe results	Develop a shared tracking document between navigator and nurse to improve coordination and transparency **(Develop stakeholder interrelationships** - Recruit and cultivate relationships with partners in the implementation effort)
*Theme 3: difficulty with identification, documentation, and enrollment of students with asthma at the beginning of the school year*	BACK program’s relationship with partnering schools is unclear	Organizational perspective- leadership	Define relationships with schools - utilizing localized and school-specific information on program materials (**Develop stakeholder interrelationships** - Recruit and cultivate relationships with partners in the implementation effort )
	School nurses are very busy at start of school year managing multiple competing priorities	Organizational perspective- burden	Navigators offered to partner with school nurse to extend support to all families of children with asthma (not just those enrolled), particularly to collect data on asthma control (asthma intake forms) to identify eligible students for BACK. (**Develop stakeholder interrelationships** - Recruit and cultivate relationships with partners in the implementation effort)
	Families lack awareness and knowledge of the BACK program	Participant perspective and characteristics- knowledge and beliefs	Adapt the program website to explicitly identify the BACK program and participating schools (**Develop educational materials** - Develop and format manuals, toolkits, and other supporting materials in ways that make it easier for stakeholders to learn about the BACK program)
*Theme 4: mismatches in program scope and alignment with school and family contexts*	Visit repetition for navigators and caregivers	Participant perspective- acceptability	Re-allocation of asthma education topics to more evenly spread information across visits (BACK intervention – shift of topics across visits)
	Limited program engagement from other school staff	Implementation- adopter training and support	Add high-priority school staff member/role (e.g. PE teacher) for the asthma navigator to meet with during the school year (**Network weaving** - Identify and build on existing high-quality working relationships and networks within and outside the organization, organizational units, teams, etc. to promote information sharing, collaborative problem-solving, and a shared vision/goal related to implementing the innovation*)*
	Strict eligibility criteria meant scope of program smaller than initially anticipated	Organizational perspective and participant perspective	Add navigator-led “Lunch bunch” group visits for any student with asthma, regardless of eligibility, to offer additional education (**Develop stakeholder interrelationships** - Recruit and cultivate relationships with partners in the implementation effort)

*Note:* *Waltz, T.J., Powell, B.J., Matthieu, M.M. et al. Use of concept mapping to characterize relationships among implementation strategies and assess their feasibility and importance: results from the Expert Recommendations for Implementing Change (ERIC) study. Implementation Sci 10, 109 (2015). https://doi.org/10.1186/s13012-015-0295-0.

With school participation in the BACK program hinging on the school nurse, we found it important to consider and address how best to promote and facilitate school nurse engagement (Theme 2). School nurses and asthma navigators felt that the second year of the program would be less “clunky” as approvals and processes were already set up and familiarity with the program increased over the school year. However, communication and transparency challenges still need to be addressed. School nurses recommended that improvement in role clarity would continue to be important for all school nurses, but particularly those schools that are newly implementing BACK. Using the ERIC strategies of “Support Clinicians” and “Support stakeholder inter-relationships” [27], we adapted the program introduction for school nurses to clarify their role, offer clear distinctions between expectations for asthma navigators and school nurses, and to emphasize program benefits. To improve transparency, coordination, and communication, asthma navigators created a data sharing dashboard so nurses can clearly see which of their students are enrolled in the program, the timelines for visits with students and caregivers, and track any asthma-related updates based on their assessments of the child’s asthma and discussions with family. Lastly, we plan to identify a regional school nurse champion for each region receiving the BACK program who can help to share information, be a resource, and promote engagement from other school nurses.

To improve recruitment and enrollment processes and reach, adaptations were launched to clearly define program relationship with schools, support school nurse processes for managing students with asthma, and increase program visibility (Theme 3). The BACK program sought to clearly define relationships with specific schools and school districts – this included offering localized and school-specific informational materials including contact information for the responsible school nurse. Additionally, we developed scripting for asthma navigators to help parents understand school-navigator partnerships, so the program feels less like a separate entity. We relaunched our study website with additional information about asthma, the BACK program, and what participation entails for families while also offering information about actively involved schools and partnerships. One benefit of these defined partnerships was for asthma navigators to help support school nurse outreach to families of students with asthma and collection of asthma care plans and intake forms; this will both alleviate the workload burden of school nurses and support student enrollment.

To promote program fit and acceptability among school nurses and other school staff, our team has adapted our existing BACK-E implementation strategies of “network weaving” among schools and communities to include an additional visit between the asthma navigator and a high-priority member of the school staff (e.g., outdoor recess monitor). Additional expansion of the BACK team to include UAPs or nurse supervisors may occur based on district and external school health system infrastructure (i.e., nurse supporting multiple schools). To expand the scope and reach of the BACK program, schools have the option to host “Lunch Bunch” and group inhaler visits to further support all students with asthma who may benefit from additional education. Based on asthma navigator bandwidth, school nurses will also have the option to refer students who frequently visit the health office for asthma-related needs but who were not initially evaluated as being eligible.

## Discussion

In order to operationalize our adaptive implementation strategy of “adapt and tailor to context,” this rapid qualitative analysis engaged key implementation partners and intervention recipients to understand contextual successes and challenges from the first year of the BACK program. These school nurse (adopter), asthma navigator (implementer), and caregiver (program recipient) perspectives are critical to help us understand how the BACK intervention and its implementation strategies align with PRISM contextual domains. Specifically, we observed challenges related to school nurse engagement and communication regarding how asthma management roles and responsibilities are shared, identification and enrollment of students with asthma, and program scope and alignment with relevant contexts. If these challenges were not addressed by program adaptation for subsequent school years, it would likely threaten many program implementation outcomes, including feasibility, acceptability, adoption, and our primary outcome of reach. Importantly, participants saw great potential benefits in terms of the likely effectiveness of BACK for participating students and families; however, many hoped for expanded impacts with more of the students in their schools also gaining some benefit in terms of general asthma education.

Challenges experienced by the BACK program are consistent with those identified when evaluating other school-based health programs that have also reported issues with school nurse/staff competing priorities, contextual characteristics, and breakdown in communication and coordination among school staff implementers and researchers [28–30]. Our findings support challenges identified in a study examining school nurse perspectives on school-based telehealth for asthma care that pointed to insufficient time and challenges to engage caregivers as substantial barriers to implementation [31–33]. Ours and others’ studies demonstrate that although school nurses may recognize benefits of school-based health programs, their competing priorities and responsibilities may limit their involvement and level of commitment and participation [31,32].

Our work builds on prior dissemination and implementation science research by systematically utilizing the PRISM and RE-AIM frameworks to understand factors influencing program integration in schools, inform program adaptation, and iteratively evaluate and refine the BACK program [34–36]. Our approach incorporated qualitative assessment across diverse implementation partners in school settings and used rapid qualitative analysis for time-dependent program refinement. Others have utilized quantitative ratings as well as qualitative findings to guide adaptations [34,36]. This study reinforces the usefulness and fit of a rapid methodological approach when timelines are contracted and informed iteration requires a rapid-turn-around for data analysis. Rapid analysis, specifically the review of interview summaries and populating of matrices occurred concurrent to data collection allowing multiple qualitative research phases to happen simultaneously. Our use of rapid qualitative analysis enabled our research team to inform adaptations that address time-sensitive challenges identified by program implementers and recipients. The use of PRISM-guided matrices for this rapid qualitative analysis is in line with others’ recommendations [23,37–39]. Our work highlights the utility of incorporating a rapid qualitative approach, particularly for studies utilizing robust deductive models or frameworks and requiring a faster pace of implementation or bound by external time constraints.

While rapid qualitative methods are a rigorous scientific method it is unlikely that schools will use this approach to support an “adapt and tailor to context” strategy for future BACK implementation due to a lack of means and resources. However, our approach did intentionally mirror processes that are used for adapting programs in school settings. For example, school leaders and nurses often review their menu of programs offered annually and determine the utility of their ongoing use and necessary modifications. Thus, this approach of adapt and tailor to context informed by rapid qualitative data is a scientifically rigorous approach utilized in this trial as an implementation strategy, and it is has less intensive analogues in the current annual review processes used in schools and other real-world settings.

The role of an adaptive implementation strategy, such as the “adapt and tailor to context” strategy, allowed us to refine our implementation strategy “package” to better fit context. Proctor et al. have described that it is ideal to present multiple implementation strategies as a “package” that guides implementers on “how a given innovation is to be enacted” [40]. Deploying adaptive implementation strategies in this hybrid type 2 implementation-effectiveness trial has been highlighted as an important advance to accelerate the speed of translation while maintaining rigorous attention to fidelity to the core functions of the intervention and implementation strategies to be tested [13]. While program and implementation adaptations were informed by our qualitative findings and are broadly applicable across regions, many of these adaptations offered further opportunity for tailoring to local contexts. For example, our “adapt and tailor to context” strategy allowed us to refine our “network weaving” implementation strategy in the BACK-E strategy package by offering schools a “menu” of options to engage school staff and promote broader program awareness. Schools were able to tailor to context by deciding which options were selected, how they were delivered, and by whom. Others have also utilized similar approaches to “adapt and tailor to context” to modify existing implementation strategy packages during a hybrid implementation-effectiveness trial [41,42]. In particular, Cordasco et al. note this is particularly important when scaling up to a relatively large number of settings with different characteristics from initial study settings [41]. As the science of adaptation continues to evolve, it is intriguing to further consider when and how to deploy “adaptive implementation strategies” to proactively review contextual data and adapt the specific strategies that no longer fit to context.

In this work, we identified key PRISM contextual factors posing a challenge to certain core functions of BACK. For example, school nurse engagement and communication are central to bridging the gap between schools and the asthma navigator and is therefore essential for fulfilling two core BACK intervention functions: 1) assessing asthma control and 2) ensuring collection of asthma care plans and medications for use in school. The beginning of the school year has proven challenging as school nurses balance competing priorities, and there is an overall lack of program familiarity from families, both of which contributed to issues associated with the core function of identifying students with asthma and enrolling eligible students.

For this project and other implementation trials, program evaluation and refinement must occur amid feasibility constraints from the implementation settings. The nature of BACK program intervention timing and the cadence of the school year calendar make ongoing program evaluation and refinement time-dependent. Data collection and analysis timelines had to balance team capacity for conducting research activities, BACK program and academic calendars, and pragmatic timing of interviews to ensure participation yielded sufficient and meaningful data. We chose to conduct school nurse interviews in the winter as this is a less-busy time of year for them; while some school nurses had limited interaction with the program and were therefore unable to comment on all of the components of the BACK program, they were able to meaningfully contribute perspectives on beginning of the year activities where their roles and expectations are more pronounced. Asthma navigator and caregiver interviews required comprehensive start-to-finish discussions of the BACK program and were therefore conducted at the end of the school year. The window between the end of family participation in early June and the start of school and associated student identification and enrollment in August leaves minimal time to complete data collection, analyze interview data, and work with the research team to decide on program adaptations to roll out in the following school year. These decisions regarding the timing of data collection across multiple program roles are important for programs to consider.

### Strengths and limitations

This study has both strengths and limitations. Our inclusion of both participant and partner perspectives including caregivers (recipients of BACK), asthma navigators (implementers), and school nurses (staff adopters), and use of PRISM to guide interviews and analysis offers a holistic view of the BACK program and is an example of using multi-level input to inform the development of comprehensive adaptations. Child perspectives were not included due to burden, logistical challenges, and study scope; caregiver and navigator interviews served as a proxy to provide insight into the child’s experiences during 1–1 education sessions in the school setting. Also, our rapid qualitative approach allowed us to conduct a rapid-turn-around evaluation of BACK with a focus on identifying program contextual successes and challenges of implementation that are important to operationalize the adaptive implementation strategy of “adapt and tailor to context.” Our rapid qualitative approach also confers limitations. This rapid approach does not allow for inductive findings nor is it well suited for identifying nuances in the data typical of traditional qualitative approaches. Another study limitation is resultant of variable levels of study participation by region with few school nurses from the LAV and few caregivers from Weld/Morgan participating in interviews. While we did not conduct a comparative analysis at this stage, results from the Pikes Peak region may be more heavily represented as this region had the largest samples across participant types. Variation in sample size is likely due to a combination of factors including region population, number of participating schools, number of enrolled students, navigator comfort with recruitment, and delays in program start-up due to staff turnover. Lastly, despite substantial efforts to recruit Spanish-speaking caregivers, we were unable to complete any interviews with this group, limiting our ability to speak to any differences in experience for these families.

Focusing on advancing science on the pace of implementation, this manuscript highlights the benefit of leveraging natural pauses in implementation, such as summer break for schools, to adapt and tailor to context. The findings from this initial 2-to-3-month rapid evaluation and planning cycle identified key challenges and a range of participant recommendations to operationalize the research team’s use of the “adapt and tailor to context” strategy that will be repeated annually. The first year of the BACK program has conferred several benefits to students with asthma, their caregivers, and school nurses including improved self-management, inhaler technique, and overall asthma control. However, challenges persist impacting implementation and intervention delivery. Our rapid qualitative approach and the use of PRISM and RE-AIM frameworks provided a necessary updated contextual assessment to guide our adaptive implementation strategy. This approach may be useful for other implementation trials using adaptive implementation strategies to nimbly pivot to ensure their intervention functions and planned implementation strategies are meeting their intended functions.

## Supporting information

10.1017/cts.2026.10730.sm001 Reedy et al. supplementary material 1 Reedy et al. supplementary material

10.1017/cts.2026.10730.sm002 Reedy et al. supplementary material 2 Reedy et al. supplementary material

10.1017/cts.2026.10730.sm003 Reedy et al. supplementary material 3 Reedy et al. supplementary material

## Data Availability

The datasets used and/or analyzed during the current study are not publicly available but are available from the corresponding author on reasonable request.

## Supplementary material

The supplementary material for this article can be found at https://doi.org/10.1017/cts.2026.10730.
