# Supplementary material for: Leveraging brief annual pauses in implementation: Using a rapid qualitative approach to inform iterative planning and adaptation of a school-based asthma program
Source: J Clin Transl Sci. 2026 Mar 25;10(1):e64. doi: 10.1017/cts.2026.10730 (PMC13107077; doi:10.1017/cts.2026.10730)
Supplement: Reedy et al. supplementary material 1 — Reedy et al. supplementary material [file S2059866126107304sup001.docx]

Supplemental Material 1: School Nurse interview guide

1. How do you feel the BACK program is going? *[PRISM – setting/nurse perspectives of intervention and acceptability/feasibility]*
   1. What is going well? What is not going so well?
   2. How does your experience with the BACK program compare to your initial expectations of the program?
2. Has the BACK program made it easier for you to care for kids with uncontrolled asthma? *[PRISM – setting/nurse perspectives of intervention – and PRISM – characteristics setting/nurse/ feasibility - early sustainment]*
   1. If yes, how has the BACK program made it easier for you to care for children with asthma?
   2. If no, what would make it easier for you to care for children with asthma? What changes or additions should be made to the BACK program to better support you in caring for children with asthma?
3. What impact do you think the BACK program has had on kids with uncontrolled asthma? *[PRISM – setting/nurse perspectives of intervention and perceived effectiveness]*
   1. How has the BACK program helped to identify kids with uncontrolled asthma?
   2. How has the BACK program helped to more formally assess asthma control (Asthma intake form)?
   3. How has the BACK program changed the ways in which you work with families of children with asthma?
4. How has the BACK program benefited you? Your team? The school? [*PRISM – setting/nurse perspectives of intervention and PRISM – characteristics of setting/nurse and acceptability (early sustainment)]*
   1. *Have there been any resources offered by the BACK program that you find helpful? Education, information, material resources? [implementation quality-resources for engagement]*
   2. How has the BACK program helped with asthma care in your school(s)?
   3. How has the BACK program helped with care coordination to support asthma management? How has the BACK program impacted the care coordination that you do as a school nurse? *[implementation quality-communication with HCP]*
5. In what ways do you feel the BACK program is a good fit for your school? *[PRISM – implementation and sustainability infrastructure]*
   1. What challenges do you experience working with children with uncontrolled asthma that you hope the BACK program will address?
6. How has the school introduced this program to students with asthma and their families? (E.g. BACK flyer, back-to-school events, school newsletter)?  *[PRISM – perspectives on intervention – specifically perspectives on the implementation strategy of program introduction—Implementation quality]*
   1. What do you think about how this program was introduced?
   2. Can you talk me through the process for enrolling students at your schools?
7. How do you feel about the involvement of an asthma navigator? *[Implementation-Intervention Quality]*
   1. How do you feel about asthma navigators completing a social determinants of health screener with families? What impacts have you noticed as a result of completing this screener, if any?
   2. What does your communication with the asthma navigator look like? How has it been, working the asthma navigator so far? *[implementation strategy quality-school communication]*
8. Nurses at schools participating in the BACK program have been invited to participate in Communities of Learning and Practice for asthma care. These are optional to attend. Have you had a chance to attend one of these?
   1. Why did you decide to/not to attend?
9. [For school nurses randomized to the ENHANCED arm only] As part of the program, your schools will send 6 newsletters each year sharing information about asthma. How do you feel about the school sending out these periodic newsletters? *[PRISM – perspectives on intervention – specifically perspectives on the newsletters/network weaving of program with school and families—Implementation strategy quality]*
   1. How have these newsletters helped students with asthma and their families? How do you anticipate these newsletters will help with students with asthma and their families?
   2. How might these newsletters help with asthma awareness at your school (school staff/teachers/coaches)?
   3. What other ways have you connected with families or other school staff about the BACK program?
10. Have you noticed any changes to the amount of time you spend supporting children with asthma since the BACK program started?
11. Thinking about the activities of the BACK program, do you recommend your school continue these to support children with uncontrolled asthma? Why or why not?
    1. What aspects would you want to continue? How come?
    2. What aspects would not want to continue? How come?
    3. Are there any incentives/metrics for school nurses/school health that the BACK program is helping you to meet? Which ones? *[PRISM – external environment]*
    4. Thinking about the future sustainability of the program, are there things that would help the BACK program to fit easily as part of usual school nursing care at your school? *[PRISM implementation and sustainability infrastructure]*
    5. We are working on plans to continue offering BACK after our research funding ends. What ideas do you have for sustaining the program? (fundraising, community partners, combining with other health programming, changes to the program)
