# Supplementary material for: Leveraging brief annual pauses in implementation: Using a rapid qualitative approach to inform iterative planning and adaptation of a school-based asthma program
Source: J Clin Transl Sci. 2026 Mar 25;10(1):e64. doi: 10.1017/cts.2026.10730 (PMC13107077; doi:10.1017/cts.2026.10730)
Supplement: Reedy et al. supplementary material 2 — Reedy et al. supplementary material [file S2059866126107304sup002.docx]

Supplemental Material 2: Asthma Navigator Interview Guide

1. To start us off, can you tell me about your overall experience as an Asthma Navigator this year? *[PRISM – setting/navigator perspectives of intervention and acceptability/feasibility]*
   1. What do you like most about the BACK program?
2. How has working with the BACK program compared with your initial expectations?
3. Does the BACK program seem to work better for certain schools/school nurses? Why do you think the program was a better fit for these schools? (probe for school/community/nurse characteristics) *[If additional clarification needed]* When I say fit, I mean, how well does the program align with the schools’ strengths and needs, values and culture, and schools’ priorities, for example. *[PRISM – implementation and sustainability infrastructure]*
   1. In what ways does BACK not work in certain schools/for certain school nurses? How could we change the BACK program for it to be a better “fit”?
4. In what ways has the BACK program provided you with helpful information to help take care of kids with uncontrolled asthma? (scripting, prompts, tips, checklists, training etc.) *[PRISM-implementation quality-resources for engagement]*
5. How does this program help to support children with asthma and their families? *[PRISM – setting/navigator perspectives of intervention and perceived effectiveness]*
   1. As an asthma navigator, what do you do to support effective asthma management?
   2. What additional benefit does this program offer to families? Schools/school nurses? Health care providers?
   3. Can you tell me about what went well and what didn’t go well in your communication with students/families? Providers? School nurses?
   4. [ENHANCED ONLY] As part of the program, schools were supposed to send out 6 newsletters sharing information about asthma. Do you know if these were sent out? *[PRISM – perspectives on intervention – specifically perspectives on the newsletters/network weaving of program with school and families—Implementation strategy quality]*
      1. What impact do you think these newsletters had on students with asthma? What about the larger school community?
      2. How were these newsletters distributed to families (modality)?
6. What concerns do you have about the BACK program? *[PRISM – setting/navigator perspectives of intervention – and PRISM – characteristics setting/navigator/ feasibility - early sustainment]*
   1. What challenges did you experience this past year?
   2. What difficulties do you anticipate in the coming years?
   3. What are some of the things you don’t like about this program? What changes should be made to address these?
7. What kinds of students/families have been engaged in the program? Has the BACK program reached those that need it? Do you feel students/families engaged in BACK are representative of the voices/people in the region you serve? [*Reach*]
   1. Who do you think the BACK program has not been able to engage? What could we do to better engage them?
8. As part the BACK program, we ask asthma navigators to meet with students/caregivers 3 times a year to teach asthma management skills and to coordinate asthma care needs. *[Implementation-Intervention Quality]*
   1. Can you tell me about how these visits went? How do you think families feel about these visits? (acceptability)
   2. In what ways are these visits helpful for families?
   3. What is challenging about these visits?
9. As part of the BACK program, we ask asthma navigators to serve as a linkage between families/caregivers, school nurses, and providers to coordinate asthma care and treatment plans. *[Implementation-Intervention Quality]*
   1. What did coordination look like this past year? How has it gone for you?
   2. In what ways has coordination supported asthma management?
   3. How engaged or accessible are these different groups (families, school nurses, providers)?
   4. What has been challenging about this care coordination component of the program?
   5. What are some ways we could improve this process?
10. As part of the BACK program, we ask asthma navigators to work with families to complete a social needs screener to assess their needs and connect them with available community resources. *[Implementation-Intervention Quality]*
    1. Overall, how did these screenings go? How do you think families feel about completing these screenings?
    2. How do you think these screenings and the referrals to resources impact asthma management for the children you support?
    3. What is challenging about screening for family’s social determinants of health concerns or connecting them to resources?
    4. What are some ways we could improve this process?
