# Supplementary material for: Leveraging brief annual pauses in implementation: Using a rapid qualitative approach to inform iterative planning and adaptation of a school-based asthma program
Source: J Clin Transl Sci. 2026 Mar 25;10(1):e64. doi: 10.1017/cts.2026.10730 (PMC13107077; doi:10.1017/cts.2026.10730)
Supplement: Reedy et al. supplementary material 3 — Reedy et al. supplementary material [file S2059866126107304sup003.docx]

Supplemental Material 3: Caregiver Interview Guide

1. Can you tell me about your experience participating in the BACK program? *[PRISM – setting/caregiver perspectives of intervention and acceptability/feasibility]*
   1. How did your experience with the BACK program compare to your initial expectations?
   2. What do you think would make the BACK program better?
2. What made you want to participate in the BACK program? *[PRISM – setting/caregiver perspectives of intervention and acceptability/feasibility]*
   1. What benefit did you hope the program would have on your child/family?
   2. What about the BACK program appealed to you?
   3. What influenced your decision to participate in the program? (i.e. time commitment, trust in the program/staff, school nurse recommendation)
3. How has your child’s asthma or asthma management changed since starting the program? *[PRISM – setting/caregiver perspectives of intervention and perceived effectiveness]*
   1. Probes: school absences, ER visits, knowledge about asthma and how to manage it
   2. After participating in the program, what about asthma management has become easier?
   3. What do you still find difficult with asthma management?
4. How easy or difficult was it for you to participate in the BACK program? *[PRISM – implementation and sustainability infrastructure, caregiver perspectives of intervention and feasibility]*
   1. What aspects of the BACK program made it easy for you to participate in the program?
   2. How did you find out about the program? Is there a better way for parents to learn about the BACK program?
   3. How well do you think BACK fit into your and your family’s lives?
5. Were you able to complete all three visits with the asthma navigator? [*Fidelity*]
   1. **If yes**, what helped you to stay or kept you in the program?
   2. **If no,** can you tell me the reasons for not completing the visits?
      1. Was there anything the school nurse or asthma navigator could have done to help you complete all the visits or stay in the program?
6. A big part of your participation in the BACK program involved interacting with an asthma navigator. Can you tell me about your experience with the asthma navigator? *[Implementation-Intervention Quality]*
   1. What did you and your child think about meeting with the asthma navigator to learn more about asthma self-management?
   2. How did you feel about meeting with the asthma navigator to learn more about managing asthma?
   3. What kinds of things did you discuss with the asthma navigator as part of the program? (i.e. asthma health screening, SDOH screening, training, SDOH referrals)
   4. What did you like about the visits with the asthma navigator? What didn’t you like about these visits?
7. Part of the asthma navigator’s role is to serve as a linkage between you (parent/caregiver), your child’s healthcare providers, and school nurses to help with communication and coordination related to asthma. Can you tell me how the asthma navigator helped with coordination on behalf of your child? *[Implementation-Intervention Quality]*
   1. What impact, if any, did this coordination have on your child’s asthma?
8. As part of the BACK program, our asthma navigators complete a social needs screener to assess parent/caregiver needs every 3 months and connect students and families with available resources to support identified needs. How did these screenings go for you? *[Implementation-Intervention Quality]*
   1. Through the screening, did you identify any needs? If so, were you connected with any resources? If so, how did it go? Did you get what you needed for your family?
   2. How did you feel when the asthma navigator was completing the screening tool?
   3. What concerns or drawbacks, if any, did you have about the BACK program screening for social needs?
   4. How did this part of the program affect your child’s asthma?
9. Would you recommend the program to others? Why/why not? *[PRISM –caregiver perspectives of intervention and early sustainment]*
10. Schools are working on plans to continue offering BACK after our research funding ends. What ideas do you have for sustaining the program? (fundraising, community partners, combining with other health programming, changes to the program) [*Sustainment*]
    1. What would you want this to look like if it wasn’t part of a study?
11. Is there anything else about the BACK program that you feel is important for me to know?
